# Negotiating commissioning pathways for the successful implementation of innovative health technology in primary care

**DOI:** 10.1186/s12913-019-4477-3

**Published:** 2019-09-06

**Authors:** Gregory Maniatopoulos, Shona Haining, John Allen, Scott Wilkes

**Affiliations:** 1 Institute of Health and Society, Newcastle University, Baddiley-Clark Building, Richardson Road, Newcastle upon Tyne, NE2 4AX UK; 2 North of England Commissioning Support Unit, Durham, UK; 3 Northern Medical Physics and Clinical Engineering, The Newcastle Hospitals NHS Foundation Trust, Newcastle upon Tyne, UK; 4 Institute of Cellular Medicine, Newcastle University, Newcastle upon Tyne, UK; 5 School of Medicine, Faculty of Health Sciences and Wellbeing, University of Sunderland, Sunderland, UK

**Keywords:** Commissioning, Decision-making, Diagnostics, Innovative health technologies, Primary care, Peripheral arterial disease, United Kingdom

## Abstract

**Background:**

Commissioning innovative health technologies is typically complex and multi-faceted. Drawing on the negotiated order perspective, we explore the process by which commissioning organisations make their decisions to commission innovative health technologies. The empirical backdrop to this discussion is provided by a case study exploring the commissioning considerations for a new photoplethysmography-based diagnostic technology for peripheral arterial disease in primary care in the UK.

**Methods:**

The research involved an empirical case study of four Clinical Commissioning Groups (CCGs) involved in the commissioning of services in primary and secondary care. Semi-structured in-depth interviews (16 in total) and two focus groups (a total of eight people participated, four in each group) were conducted with key individuals involved in commissioning services in the NHS including (i) senior NHS clinical leaders and directors (ii) commissioners and health care managers across CCGs and (iii) local general practitioners.

**Results:**

Commissioning of a new diagnostic technology for peripheral arterial disease in primary care involves high levels of protracted negotiations over funding between providers and commissioners, alliance building, conflict resolution and compromise of objectives where the outcomes of change are highly contingent upon interventions made across different care settings. Our evidence illustrates how reconfigurations of inter-organisational relations, and of clinical and related work practices required for the successful implementation of a new technology could become the major challenge in commissioning negotiations.

**Conclusions:**

Innovative health technologies such as the diagnostic technology for peripheral arterial disease are commissioned in care pathways where the value of such technology is realised by those delivering care to patients. The detail of how care pathways are commissioned is complex and involves high degrees of uncertainty concerning such issues as prioritisation decisions, patient benefits, clinical buy-in, value for money and unintended consequences. Recent developments in the new care models and integrated care systems (ICSs) in the UK offer a unique opportunity for the successful commissioning arrangements of innovative health technologies in primary care such as the new diagnostic technology for peripheral arterial disease.

## Background

Despite the growth of studies examining the process of implementing technologies in healthcare, there has been limited research on the decision making processes (i.e. pre-implementation assessment criteria) surrounding the commissioning of innovative health technologies in practice [1]. This oversight is somewhat paradoxical given current policy pressures towards ‘evidence-based policy-making’ and ‘evidence-based decision-making’ [2] and the push for adoption of new technology in healthcare. In particular, understanding how decisions are made by those commissioning healthcare services still remains unclear.

Over the past decade, the role of commissioning has become increasingly important to the health system in England. Commissioning is a decision making process by which public services are planned, contracted and monitored to meet population needs. It involves much more than procuring services and managing transactional issues with activities ranging from the health needs assessment for a population, through the clinically based design of patient pathways, to service specification and contract negotiation or procurement, with continuous quality assessment [3]. The Health and Social Care Act (HSCA) 2012 which is the legislative basis for the current NHS structure led to significant changes in the organisation of the NHS. It brought in a new commissioning environment in which competition, patient choice and integration of services each play a more prominent role. Most of the National Health Service (NHS) commissioning budget is now managed by clinical commissioning groups (CCGs). At a national level, NHS England is responsible for the commissioning of services outside the remit of clinical commissioning groups, in particular specialized services, primary care and some public health services [4].

In the context of health services research this lack of attention could be related to the timing and the methodological difficulties (i.e. negotiating access) researchers may have in capturing the decision making process for commissioning innovative health technologies. Typically, empirical studies have been carried out retrospectively at some chronological distance from the initial commissioning negotiations, the outcome of which underpins the identification, assessment and decision making criteria surrounding the implementation of care pathways. Indeed, in most cases the process of commissioning is only briefly introduced prior to the implementation phase where it becomes subject to economic forms of analysis (i.e. commissioning as a technical or operational process) where the assumption is that commissioning is the outcome of formal planning and economic assessment procedures which seek to maximise the value of resources and improve the health and wellbeing of the community [5]. More recent sociological approaches view commissioning as a relational process, driven by the micro-politics of the organisational setting, competing agendas, interests, priorities, demands and personal inclinations to build a persuasive, compelling case [5–7]. A distinguishing feature of these approaches is their emphasis on the social dynamics and power relations within which commissioning decisions become enacted over time. In so doing, these sociological explanations highlight the various contingencies and uncertainties surrounding decision-making as a complex process of conflict, negotiation and alignment [8]. Drawing upon Strauss’ negotiated order perspective [9, 10], we view commissioning decision-making as an outcome of contingent negotiations among different social actors who collectively negotiate the formal and informal ‘rules’ that shape the delivery of healthcare services. The negotiated order perspective provides a means to understand the processes involved in commissioning decision-making by exploring the social structures and conditions that shape those processes. In line with such ideas we argue that commissioning should be understood as an emergent and contingent process in that it is constantly defined and redefined across multiple contexts. As such, both the technical and organizational dimensions of commissioning decision making tend to be characterized by high degrees of uncertainty concerning such issues as prioritisation decisions, patient benefits, clinical buy-in, value for money and unintended consequences [11].

This paper explores the process by which commissioning organisations make their decisions to commission innovative health technologies. The empirical backdrop to this discussion is provided by a case study exploring commissioning considerations for a new and fast diagnostic technology (utilizing multi-site photoplethysmography; MPPG) [12–14] for peripheral arterial disease in primary care, a diagnostic innovation for the treatment of vascular disease. Peripheral arterial disease (PAD) affects approximately 20% of patients aged ≥60 years [15] and is a lens to view additional cardiovascular pathology including myocardial infarction and stroke risk [16]. Currently, there is a high degree of variation related to the availability and delivery of PAD services across primary and secondary care. The new MPPG-based PAD device uses an automated portable diagnostic technology developed by a North East England device development team (NIHR i4i funded project: II-C1–0412-20,003) which aims to improve on current diagnostic accuracy, reduce test speed and is simple to use by a range of health care practitioners in primary care. It is aimed to be used at the first patient contact and to identify patients that need either reassurance, primary prevention of cardiovascular risk factors or onward referral for specialist intervention [15] and ultimately to reduce unnecessary referrals [17]. This is particularly important when current guidance is a default referral strategy to a vascular surgeon if there is doubt about the diagnosis [18, 19]. At present, the ankle brachial pressure index (ABPI) test (ratio of the ankle to brachial systolic blood pressure as usually measured by sphygmomanometer and hand-held Doppler ultrasound probe) is widely used and by a diverse range of practitioners (in the community and hospital setting) to screen asymptomatic patients, diagnose patients with clinical symptoms, and to monitor patients who have had radiological or surgical intervention [20]. However, as many as one-third of patients at risk are currently going undetected [21] when primary prevention measures could have a significant impact upon future morbidity and mortality. In the context of primary care, as many as one in five patients in the general practice setting may have peripheral arterial disease [22] and the question of testing for such disease comes into sharp focus particularly when new technology such as the MPPG-based PAD device may make this a real possibility and also serves as a window on general cardiovascular health. Here, we explore the process by which commissioners make their decisions to commission a pathway for the new diagnostic technology in primary care practice.

## Methods

Semi-structured in-depth interviews (16 in total; see Table 1) and two focus groups (a total of eight people participated, four in each group) were conducted between July 2015 and September 2016 across four localities in one geographical region in England. The semi-structured interviews were conducted with commissioners working across four clinical commissioning groups in a mixed urban and rural area. Two focus groups conducted with one CCG in a rural area, aimed to capitalise on the interactions between a group of senior commissioners providing a balance with the semi-structured interviews [23]. A topic guide, informed by previous studies on commissioning [24] was developed to structure the interviews and focus group, the same guide used for both, to explore the decision making process of commissioning by participants. The topic guide was designed to encourage participants to reflect on successful and unsuccessful practices for commissioning in the context of innovative health technology and to cover perceptions of (i) the organisational structures, processes, relationships, barriers and facilitators related to commissioning and (ii) the relative influence of policy drivers, relationships with providers and external influences.

**Table 1:** List of participants

Organisation	Number of interviews	Interviewees
CCG Site 1	5	Senior Manager × 2
		Medical Director/GP
		Clinical Director/GP
		Head of Finance
CCG Site 2	4	Senior Manager/GP ×2
		Senior Commissioning Manager × 2
CCG Site 3	4	Clinical Director
		Senior Commissioning Manager × 3
Clinical Network	1	Senior Manager
NHS	2	GPs
Focus groups		
CCG Site 4		Senior Clinical Officer
		Clinical Director
		Senior Commissioning Manager ×4
		Locality Manager ×2
Total	24	

Participants were purposively selected according to their role and involvement in commissioning services in primary and secondary care, on the recommendation of members of an external research advisory steering group set up for the purpose of the study. Participants included (i) senior NHS clinical leaders and directors (ii) commissioners and health care managers across CCGs and (iii) local general practitioners (GPs). The steering group was formed to provide specialist advice around primary care commissioning, review interim findings and assist with the evaluation of the outputs of the project. The steering group met every 6 month and included (i) an NHS clinical director and (ii) two senior commissioning managers/GPs.

Potential interviewees were sent an email invitation which outlined the aims and objectives of the study. Those agreeing to participate were also invited to recommend additional candidates for interview. Individuals who agreed to participate in the study were provided with information sheets in advance, and consent forms were signed prior to the start of the interviews. All interviews and focus groups were digitally recorded, anonymized and transcribed in full. Interviews were typically around 60 min in length and conducted on an individual, face-to-face basis. Each focus group was approximately 90 min long and conducted at the workplace of the participants. Two researchers (GM and SW) conducted the fieldwork. Many interviewees had multiple roles both as commissioners and providers of primary and secondary care services and therefore opinions expressed often represented their experiences from both areas of practice.

### Data analysis

Drawing upon an interpretative approach interviews were analysed inductively, without the aid of a software programme. Data transcripts were read through to familiarise with content and were then subjected to thematic analysis [25]. Core categories and themes were identified for each participant and then compared within each case. Two members of the research team (GM and SW) undertook the analysis of interview data and emerging categories. The themes were then reviewed and discussed within the wider project team meetings with any discrepancies resolved through this process. The results were also validated with the steering group which was formed to provide guidance on the research design and review interim findings. In this paper, all participants have been anonymised.

### Ethics

A positive ethical opinion was obtained from Newcastle University Faculty of Medical Sciences Ethics Committee (ref: 00831/2015). NHS Research Ethics approval was not required for this study as per the Health Research Authority guidance.

## Results

Analysis of the data generated five broad themes relating to commissioning a pathway for the new type of PAD diagnostic technology in primary care practice: 1) managing competing priorities 2) valuing evidence 3) assessing value for money 4) cross-organisational collaboration 5) and negotiating commissioning pathways. These themes represent some of the factors shaping the decision-making processes surrounding the commissioning of the new diagnostic technology in primary care rather than linear criteria to determine such decisions. Indeed, the overall context in which the complex process of commissioning and how decision-making takes place remains both uncertain and fluid.

### Managing competing priorities

Participants highlighted the important role of priority setting in commissioning a pathway for the new diagnostic technology in primary care practice. With limited resources, improving the quality and outcomes of services for the local population was considered to be a key factor shaping commissioning decision making.

*It needs to link with our priorities because we have a finite amount of resources … We can't afford to fund things that are doing well, to fund it even further* (Senior Commissioning Manager, CCG Site 3)

It was acknowledged that the drive for change may come from multiple sources and informed by both internal (expert advice from GPs/clinicians) and external (clinical guidelines) factors.

*… It could be that our GPs say to us, "There is a big issue around peripheral vascular disease, people aren't being managed properly." It could be secondary care clinicians who come to us and say, "There is a new technology that we think would improve the patient pathway that we think we should have a look at." It could come from NHS England or the Health and Wellbeing Board who see that there is a big issue around peripheral vascular disease in England … NICE (National Institute for Health and Care Excellence) might produce a tag or a guidance around it. So it could be any of those things …* (Senior Manager, CCG Site 1)

Commissioners suggested that a wide variety of information sources are used to inform their prioritisation decisions. This includes intelligence from local public health teams and comparative benchmarked information from national data in order to identify unwarranted variation as a mechanism to releasing resources for re-investment in higher-value healthcare for local patients and populations.

*We use things like the Atlas of Variation, other benchmarking tools that allow us to see where we are in relation to other organisations… And on the basis of that, we develop a set of priorities. We normally set our priorities three and five years in advance and we would then look at something, and idea or innovation that was coming, and then say, "Right, okay. Does that link with any of our priorities?" If it didn't link with any of our priorities, we would have a look at it still and we would say, "Right, okay. Is it something that we maybe haven't thought of before? Is it something that we weren't aware of?"* (Senior Commissioning Manager, CCG Site 3)

These comparative measures could operate as forms of accountability for commissioners by justifying particular decisions. In so doing, they play a key role in framing future investment/disinvestment decision-making. Their efforts involve the construction of a ‘like for like’ comparison between the health outcomes of local populations.

*The very start of the process is we look at health needs of local population. We look at how we compare and benchmark to other organisations, and do we have a particular outcome or adverse outcome either from a health point of view or from an unmet need point of view. So we really do look at what the need is of our local population. How do our patient outcomes compare to other patient outcomes?* (Senior Commissioning Manager, CCG Site 2)

A number of participants pointed to the benefits of being able to draw upon the support from national bodies such as NICE and NHS England, but there was evidence of a tension between national priorities and the need to meet the rapidly changing needs of local populations. Often local conversations could complement national evidence and information from other localities.

*I would be involved in that discussion about whether something is a priority area for us. If it is a priority area for us and we agree that we’re going to put it in as part of our commissioning intentions for next year, over the course of the year there’ll be development of a specification for the service, which would include quality standards, which is my particular part of it.* (Focus Group 2, Clinical Director, CCG Site 4)

The need to respond to priority areas mean that CCGs are frequently required to make difficult prioritisation decisions according to the needs of their local populations. Given the tension between national and local agendas for some participants competing priorities could hinder commissioning a pathway for the new diagnostic technology in primary care.

*I think competing priorities are probably the biggest […] and everything’s priority isn’t it?* (Senior Manager, Strategic Clinical Network)

The tensions between national and local agendas and competing priorities, accompanied by growing financial pressures on the NHS raised issues and concerns about the ways in which current commissioning practices based on measurement of activity - such as waiting times and the number of patients treated - are organised, managed and performed. Traditionally, most commissioning processes in healthcare have tended to pay providers for particular tasks and activities often within a set period of time, rather than for the delivery of agent’s achievement of specific results, or outcomes.

*It’s very complex; the whole thing is incredibly complex and you wouldn’t set up commissioning like this if you were starting from scratch, but we’re working in a complex environment and we just have to find our way around it.* (Clinical Director/GP, CCG Site 1)

In summary, commissioning decision-making becomes subject to potentially conflicting criteria under conditions of uncertainty and competing priorities. By negotiating between competing agendas, power relationships and personal choices commissioners strive to build persuasive, compelling arguments to inform local commissioning decisions.

### Valuing evidence

Participants highlighted the role of evidence in commissioning a pathway for the new diagnostic technology. It was stressed that any commissioning decision should be informed by a variety of different evidence sources and expert involvement to ensure that effective decisions are made for local populations.

*The pathways in totality should be evidence-based pathways. Therefore, anything that’s within that would need an evidence base to supports its introduction…Does the introduction of this technology equate to high quality? Is it safe? Will it actually improve the patient experience because it’s done closer to home? Does it join-up with the rest of the system, because there’s no point introducing a technology in general practice if it isn’t a technology that’s accepted by secondary care as part of a process.* (Senior Manager, CCG Site 1)

Best practice guidelines and research were described as potential sources of information commissioners drew on when making decisions, some of which they sought out, while others were imposed upon them. Much of the other evidence that is relevant to commissioning is more likely to be generated by commissioners themselves (e.g. assessments of need) and providers of services (e.g. service user feedback and outcomes monitoring/evaluation of outcomes).

All the commissioners in the participating CCGs stressed that their support for the new diagnostic was on account of the potential benefits it would have for patients. Agreeing a shared understanding of the value added to the clinical pathway was considered to be important for developing a business case to support the commissioning of the new diagnostic technology.

*With any new services, whether it is a technology service or any new pathway, you would be looking to say, "What is new about this that adds value to the existing pathway?" Does it improve the quality of care? Does it improve the patient experience? Is it more economical? What is the hook? Because clearly there has got to be a reason why you would adopt something, so you have to be very clear about what that reason is… it has got to be two things. It is a balance between improving the quality of care but also the efficiency. So is it value for money?* (Senior Manager/GP, CCG Site 2)

In this context, any decision to commissioning the new diagnostic technology would be subject to commissioners gathering evidence and making a business case: weighing up cost savings and quality improvements. Different types of information and analytical resources were considered to be important in shaping the development of a business case for commissioners incorporating both explicit and tacit knowledge channels. This included clinical, ethical, organisational, technological and economic considerations (e.g. clinical and financial evidence), as well as subjective local judgments (e.g. how difficult something is) in relation to local implementation and adoption issues. These factors are not independent of each other, and indeed could be mutually reinforcing and/or conflicting.

*Once it fits with that [within priority areas], we would then have a look at what the innovation is, have a look at the evidence, see how it links with any other things that we're doing. What stage is it at? Is it at trial stage? Has it been used anywhere? Has it been implemented? And then if we agree that it's something we want to look at further, we would then work it up. So it would be worked up in terms of a business case… We would then decide, what is the case? Is there a clinical case there? How strong is the evidence base? What sort of outcomes will we be looking to achieve? Will it address the priorities we've identified at the start of the process? What sort of risks relate to it? What is the degree of difficulty? What sort of time frame are we talking? Is it something that could be implemented quite quickly? Would we recommend a trial? So, would we be looking at trials, or have trials already taken place and we would simply be looking to move to implementation?* (Senior Commissioning Manager, CCG Site 3)

Apart from the importance of structures, resources and formal processes shaping decision-making, participants stressed also the importance of ‘soft’ skills of clinical leadership to support innovation and service improvement through new technologies.

*It would need to have been championed clinically because we are a clinically-led organisation. Before it gets to the executive, that value would then have been tested out throughout our financial colleagues and our performance colleagues and various interactions throughout the organisation. When it reaches the executive, it has been sense checked by a number of different individuals with different skill sets and different perspectives on a case that are all then sitting there saying, “The ducks line up.”* (Senior Manager, CCG Site 1)

The implications of these insights for commissioning decisions for the new diagnostic technology are that important factors to consider include levels of complexity and uncertainty, as well as the expected controversy and impact of decisions. Commissioning decisions take place in a context characterised by high levels of uncertainty, where different evidence sources and the decision criteria are negotiable and indeed openly and covertly contested.

### Assessing value for money

Although improvements in patient care were considered to be important for commissioners, particular emphasis was also placed to the extent to which implementing the new diagnostic technology was value for money.

*From a CCG perspective… we would look at the cost. So generally as a rule, as a CCG, things are looked on better if they are value for money, if they are looking at releasing resource so we can invest that resource in to other areas of health care…the clinical side of things always outweighs the cost side of things. But at the same time there is a ceiling to what we can pay.* (Senior Commissioning Manager, CCG Site 2)

In this context, the financial evidence would seek to outline the proposed investment in terms of the affordability of the proposed pathway, the clinical benefits and the overall value for money. In line with economic arguments associated with new diagnostic technology participants highlighted the need to demonstrate cost-benefits across the wider health system.

*If there is something that we can demonstrate as either producing the same outcomes or indeed better outcomes for less cost or improving outcomes and numbers at the same cost then that should be part of our QIPP [Quality, Innovation, Productivity and Prevention] plan…* (Head of Finance, CCG Site 1)

Participants acknowledged that policy pressures to deliver short term cost savings meant that long term investment in patient outcomes was difficult.

*So if you can introduce something today that’s going to make a saving tomorrow, it’s much easier to account for that […]The hardest thing for a commissioner to be able to articulate is, “This will save you lots and lots of money for this particular individual for this particular cohort of patients in 20 years’ time.”* (Senior Manager, CCG Site 1)

*The unfortunate thing about the NHS is the capacity to invest to save five years’ hence is not there. You need to be able to make savings fairly quickly…* (Focus Group 1, Senior Commissioning Manager, CCG Site 4)

Moreover, financial pressures and limitations over budgets were considered to be a key barrier for commissioning a pathway for the new diagnostic technology in primary care practice making change difficult.

*So this very large budget that the commissioners have, 99% of it is allocated all the time. Because 99% is allocated already, we know it’s going to be spent on hospitals, community services and social care, you know, continuing health care. So 1% is left, it’s quite difficult to commission new stuff with tiny amounts of money…* (Medical Director/GP, CCG Site 1)

In responding to the above challenges participants discussed how CCGs are frequently required to strategically realign resources to enable the commissioning of additional services to both primary and secondary care.

*…there is no new money coming in from a CCG. So you would have to assume that what you want to commission in addition from general practice will have a reduction in cost elsewhere…I think that’s where commissioners have a problem.* (Senior Manager, CCG Site 1)

The need to legitimate value for money accompanied by growing pressures on service improvement represented a stark tension between the need for short-term investment and the longer-term requirement for service transformation and improvement.

### Cross-organisational collaboration

Commissioning of the new diagnostic would only be possible with re-configurations in clinical workflow and practices across primary and secondary care. Indeed, while the implementation of the new diagnostic was expected to demand reconfigurations across care settings, their exact form and nature was complex and unclear.

*Then what you would have to think about then is, why is that better than having the diagnostic in secondary care?… If you wanted to put a service in, it would depend where you wanted to put it in primary care. There is tons to unpick there…Do you want to move the diagnostic into a community setting or do you want to move it into a General Practice setting? Who are you expecting to administer the tool?... There is a limit to the capacity they have to take on new work. So what would the workload be if this was coming into primary care?* …*but you also have to think about, in secondary care there will be people that are already doing diagnostics of some kind for peripheral vascular disease…Do you re-deploy them?....What do you do? And actually you need to keep some of it, because of the people that you are going to look at in primary care, some of them will need to be referred and need more sophisticated diagnostics possibly? So there is something about how do you manage the system to make sure you don't close something off there that you are going to need later on. Do you see what I mean?… No it is not simple at all, not at all* (Senior Manager, CCG Site 1)

Participants acknowledged that closer collaboration between care settings is key for a potential re-configuration of a pathway for peripheral arterial disease. In particular, mutual trust between healthcare professionals in primary and secondary care was considered to be central to the successful implementation of the new diagnostic technology.

*The other thing about doing diagnostics in primary care is that you need specialists to recognise it and value it. What we can’t afford to do is have diagnostics that are not supported by specialists because they’ll do them again …* (Senior Manager/GP, CCG Site 2)

Alongside the lack of a clear pathway for the new diagnostic technology questions were raised about primary care’s capacity and resources to deliver additional services. While a shorter care pathway and fewer hospital referrals would be welcome to patients, there were opportunity costs that might put GPs acceptability in question.

*Yes, cost; the primary care saying, “Why should we use this? What is it going to do for us? Is it going to take more time to do?” Are they going to have longer appointments? Are their staff going to have to leave patients to go and get extra training? I think you’d have to have a big sell to say, “What’s the benefit of this technology? Why is it good for the patient? Why is it good for the practice? Why is it good for the CCG?”* (Clinical Director/GP, CCG Site 1)

Lack of time and capacity issues seemed to be apparent within general practice. Participants spoke about time and capacity limits, as illustrated here:

*…General Practice is under a huge amount of pressure....there is a shortage of GPs. So asking them to do ever more is sometimes not a question of money, it is a question of capacity…what incentive is there for him to take on more work, with no resource, when he could just refer the patient to the hospital and have it all done there?* (Senior Manager, CCG Site 1)

*At the moment, general practice is busy. They absolutely don’t want any extra work. I just know…They just don’t have the time to do it...* (Senior Manager/GP, CCG Site 2)

In this context, participants discussed how implementing change in primary care is not always a straightforward process.

*I think it’s probably quite complicated…because each practice is so different, there are different ways of working. You might find one practice who are really outward-looking, really engaged and change, but other practices won’t have that same attitude.* (GP 1)

The complexity of collaborative structures across primary and secondary care needs to be considered when exploring the process by which commissioning organisations make their decisions to commission innovative health technologies. Although the commissioning of the new diagnostic promised benefits over existing practice(s), its adoption also involved reconfigurations across a multi-level set of work practices that were not always easy to implement.

### Negotiating commissioning pathways

The uncertainty associated with the lack of a clear pathway for the new diagnostic technology raised also concerns about the potential commissioning re-configurations required to implement the new care pathway. Overall, participants talked about the difficulties in moving resources between different care settings.

*What would you expect to pay GPs to do that bit of the diagnostic? How would you unbundle that from the current tariff that we have with secondary care? Because at the moment the tariff we have for a new outpatient appointment includes the diagnostics. So you would have to unbundle that and say, "Okay, if it is £80 for a new out-patient appointment in second care and that includes the diagnostic, how much are you going to take out of that and give it to the doctor in primary care to do?"… That is going to be your biggest problem… So there are two issues, one is have they got the capacity to do it? And the other is can you pay them to do it?* (Senior Manager, CCG Site 1)

Given that a number of diagnostic services are currently provided in secondary care it was unclear how a potential re-configuration of commissioning arrangements from secondary to primary care could be achieved.

*It won't be financially sensible for CCGs to do that because we already pay. Why should we pay more for a GP to do it again? We already pay the Community Trust for a service. Why would we pay the GP to do it? By taking the work out of the Community Trust, it’s not going to reduce our block contract. It’s exactly the same value. Why? Why would we pay more?* (Senior Manager/GP, CCG Site 2)

The financial implications of a whole systems approach across primary and secondary care raised concerns for some of the participants. In particular, participants revealed that by moving the diagnostic into primary care with the potential of reducing unnecessary referrals to secondary care could result in a loss of income to secondary care.

*There will be a huge argument about it, because primary care want more money, secondary care don't want to lose it… So they will say, "That £80 that you gave us, only £20 of that is the diagnostic, the other £60 is administering the appointment, seeing the patient, the consultant's time, the nurse's time, the prescription that they might get at the end of it…But in primary care they will be saying, "Of that £80, if you want us to do this we want £40 of it."…And you have got to persuade primary care that it is in the best interest of patients to do that…* (Senior Manager, CCG Site 1)

*So hospitals now are much more likely to obviously look at the clinical benefits of what it is that they do, but also as likely to look at which the financial consequences are for them of participating in it as well. Do they lose money?... It always comes back to the resource.* (Senior Commissioning Manager, CCG Site 2)

Some participants considered the root of this problem as the ‘silo’ culture, where primary and secondary care organisations operate as independent units.

*There are all sorts of reasons why you would want to do that but the flow of money is a barrier to that…because they are two different organisations…There is no payment by results for primary care. It does exist in hospital. It doesn’t exist in primary care. Often that is a huge barrier…* (Medical Director/GP, CCG Site 1)

*The money, agreeing what we were going to pay GPs to do this, instead of the patient going to hospital and agreeing that. Taking the money out of the hospital budget. When money is really scarce, as it is now, nobody gives it up willingly, without a fight. So the hospital – what was the incentive for them to give up this activity that generated money for them?* (Senior Commissioning Manager, CCG Site 3)

Given the above challenges some participants argued that the healthcare economy would benefit from a more integrated approach in both the commissioning and the delivery of patient services. In particular, it was suggested that recent initiatives around the development of new care models could provide solutions to long standing commissioning barriers between primary and secondary care by removing organisational boundaries and redesigning poorly aligned financial incentives across different stakeholders which have historically prevented transformation in care [26].

## Discussion

Drawing upon Strauss’ negotiated order perspective [9, 10] this paper has explored the process by which commissioning organisations make their decisions to commission a pathway for the new diagnostic technology in primary care practice. This approach enables analysis of the negotiations that occur in commissioning decision making, as well as consideration of the interdependencies among factors shaping this process. By putting the focus on commissioning, we want to show how decisions become subject to uncertainty driven by the micro-politics of the organisational setting, competing agendas, interests, priorities, demands and personal inclinations to build a persuasive, compelling case. Instead of adopting a simple technical rationality, our study aims to understand how decision-making becomes enacted in the context of healthcare commissioning by exploring the social structures and conditions that shape those processes.

We explored these issues through a case study linked to the commissioning of a new type of diagnostic technology for peripheral arterial disease in primary care, which illustrated some of the challenges surrounding commissioning a pathway for its embedding into healthcare practice. Although the implementation of the new diagnostic promises wide-ranging benefits over existing practice(s) such as the opportunity to improve diagnostic accuracy and to identify patients that need either reassurance, primary prevention of cardiovascular risk factors or onward referral for specialist intervention, we saw how its commissioning involved high levels of protracted negotiations over funding, managing competing priorities, alliance building, conflict resolution and required reconfigurations across a multi-level set of work practices that were not always easy to implement [27].

Our evidence highlighted the important role of priority setting in commissioning a pathway for the new diagnostic technology in primary care practice [28]. However, resource limitations, competing priorities and a lack of a clear pathway could be the key challenges in the commissioning the new diagnostic technology in the NHS. Currently, there is a high degree of variation related to the availability and delivery of PAD services across primary and secondary care. In many instances PAD services are only commissioned at the end of the patient journey in the hospital setting where diagnosis and intervention (or not) usually happens. Currently, there is little evidence for formally commissioning a PAD pathway and the management of such patients appear to happen by default. This may be linked to healthcare spending priorities or to an assumption that cardiovascular risk factors associated with PAD are managed in other commissioned parts of the health system and hence only addressing the end-stage of the patient journey by commissioning hospital-based intervention services.

Further and wider evidence will always be required to assess the potential patient benefits, clinical value and cost savings for commissioning a pathway for a new diagnostic technology. Our findings suggest that most commissioners will look towards NICE (National Institute for Health and Care Excellence) and/or local clinical networks and clinical champions for guidance on commissioning decisions, particularly where the evidence is lacking [29]. It is incumbent upon these individuals and organisations to make the business case, articulate the patient benefits and demonstrate value for money that the NHS receives for its investment or realignment of services and spend. However, of particular concern among the participants was that too much was being expected too soon in terms of demonstrating a ‘return on investment’ in innovative health technology investments.

At the clinical pathway level, our data, suggests that whilst clinical champions can be important facilitators for commissioning a pathway for the new diagnostic technology, reconfigurations of work practices within and across care settings and professional groups [29, 30] to support the implementation of the new technology requires much more and remains unclear. In particular, closer cross-organisational collaboration and trust between healthcare professionals in primary and secondary care was considered to be central to reconfiguring the care pathway [31, 32]. Alongside the need for reconfigurations of work practices within and across care settings questions were raised about primary care’s capacity and resources to deliver additional services as ABPI assessment is primarily performed by secondary care commissioned community staff [33]. While a shorter care pathway and fewer hospital referrals would be welcome to patients, there were implications that might put GPs acceptability in question. Ultimately, failure to deploy accurate diagnostic technology in a primary care setting could result in unnecessary referrals of individuals without PAD or delay in access to specialist services for those that require intervention and thus more costs and poorer patient outcomes.

At an organisational level, commissioning a pathway for the new diagnostic technology in the NHS required addressing uncertainty about the effectiveness of a proven business model. From a commissioning perspective, making a case for the implementation of the new diagnostic demanded significant inter-organisational work if commissioners were to be convinced that this could be done within acceptable levels of risk financially and operationally [34]. This required removing the artificial barriers between primary and secondary care commissioning which could lead to disjointed services with variable access to diagnostic services in pathways [35]. Some of the artificial barriers articulated in this work have been quite stark. From the primary care perspective clinicians are incentivised to reduce demands upon the healthcare system and to minimise referrals. Conversely, primary care doctors often don’t have time to perform a diagnostic work-up of patients which leads to an automatic default of onward referral to specialist services. On the whole the diagnostic work-up in terms of ABPI assessment is performed by secondary care commissioned community staff. Currently there is a perverse incentive to allow all patients to progress to referral which will attract Payment by Results (PbR) tariff - the system in NHS England under which commissioners pay healthcare providers for each patient treated, taking into account the complexity of the patient’s healthcare needs [36] - income to the secondary care provider of those services.

In summary, this study reveals that the commissioning of innovative health technologies, such as a new diagnostic technology for PAD, is currently ‘messy and fragmented’ [37]. It involves high levels of protracted negotiations over funding between providers and commissioners, alliance building, conflict resolution and compromise of objectives where the outcomes of change are highly contingent upon interventions made across different care settings. Our evidence illustrates how reconfigurations of inter-organisational relations, and of clinical and related work practices required for the successful implementation of a new technology could become a major stumbling block to commissioning negotiations. In so doing, we aim to contribute to the growing area of health services commissioning by providing a more comprehensive analysis of the decision making processes surrounding the commissioning of new technologies. Learning more about how commissioners in the real world make their decisions to commission innovative health technologies is important in order to increase our understanding about the contribution that researchers and research evidence can make to policy-making.

In the light of the new care models programme across the UK [26, 38] there is now a window of opportunity to examine and evaluate the impact of Integrated Care Systems (ICSs) for the adoption of new innovative health technologies such as the diagnostic technology for PAD. This could facilitate the development of effective practical and feasible solutions for the commissioning and provision of new care pathways.

### Limitations

This study is based upon the experience of senior clinicians in commission roles and commissioning managers in a number of Clinical Commissioning Groups in one geographical region in England and may not be sensitive to the subtle variations across the UK. We also acknowledge that a potential limitation of our analysis is the focus on commissioning a single technological innovation as an example, which raises questions about the extent to which the commissioning tensions identified are common to other technological innovations in healthcare - there is certainly scope for future wider research to address these matters. Because the focus is solely on NHS commissioning the role of private health care providers of diagnostics, such as pharmacy outlets, needs also to be considered.

## Conclusion

Innovative health technologies such as new diagnostic technology for PAD are commissioned in care pathways where the value of such technology is realised by those delivering care to patients. The detail of how care pathways are commissioned is complex and involves high degrees of uncertainty concerning such issues as prioritisation decisions, patient benefits, clinical buy-in, value for money and unintended consequences. The likely successful commissioning and implementation of such technology in the NHS will depend on reconfigurations across a multilevel set of practices and its impact on patient outcomes associated with care pathways. Recent developments in the new care models and integrated care systems (ICSs), which aim to remove cross-organisational barriers/tensions in the UK offer a unique opportunity for the successful commissioning arrangements of innovative health technologies in primary care such as the new diagnostic technology for peripheral arterial disease.

## Data Availability

The datasets analysed during the study are stored on a secure server and are available from the corresponding author on reasonable request.

## Abbreviations

ABPI: Ankle Brachial Pressure Index
CCGs: Clinical Commissioning Groups
GPs: General Practitioners
ICSs: Integrated Care Systems
MPPG: Multi-site Photoplethysmography
NHS: National Health Service
NICE: National Institute for Health and Care Excellence
PAD: Peripheral Arterial Disease
PbR: Payment by Results
UK: United Kingdom
